# Associations of malaria, HIV, and coinfection, with anemia in pregnancy in sub-Saharan Africa: a population-based cross-sectional study

**DOI:** 10.1186/s12884-020-03064-x

**Published:** 2020-06-29

**Authors:** Paddy Ssentongo, Djibril M. Ba, Anna E. Ssentongo, Jessica E. Ericson, Ming Wang, Duanping Liao, Vernon M. Chinchilli

**Affiliations:** 1 Center for Neural Engineering, Department of Engineering, Science and Mechanics, Penn State University, University Park, PA USA; 2 Department of Public Health Sciences, Penn State College of Medicine, Hershey, PA USA; 3 Center for Applied Studies in Health Economics, Penn State College of Medicine, Hershey, PA USA; 4 Department of Pediatrics, Penn State College of Medicine, Hershey, PA USA

**Keywords:** Anemia in pregnancy, Demographic and health surveys, Hemoglobin, HIV, Malaria, Coinfection, Low- and middle-income countries, Iron supplementation, Sub-Saharan Africa

## Abstract

**Background:**

Malaria and HIV are common infections in Africa and cause substantial morbidity and mortality in pregnant women. We aimed to assess the association of malaria with anemia in pregnant women and to explore the joint effects of malaria and HIV infection on anemia in pregnant women.

**Methods:**

We used nationally representative, cross-sectional demographic and health surveys (DHS) that were conducted between 2012 and 2017 across 7 countries of sub-Saharan Africa (Burundi, the Democratic Republic of the Congo, Gambia, Ghana, Mali, Senegal and Togo). The outcome variables were anemia (defined as a hemoglobin concentration < 110 g/L), and hemoglobin concentration on a continuous scale, in pregnant women at the time of the interview. We used generalized linear mixed-effects models to account for the nested structure of the data. We adjusted models for individual covariates, with random effects of the primary sampling unit nested within a country.

**Results:**

A total of 947 pregnant women, ages, 15–49 y, were analyzed**.** Prevalence of malaria only, HIV only, and malaria- HIV coinfection in pregnant women was 31% (95% CI: 28.5 to 34.5%, *n* = 293), 1.3% (95% CI: 0.77 to 2.4%, *n* = 13) and 0.52% (95% CI: 0.02 to 1.3%, *n* = 5) respectively. Overall prevalence of anemia was 48.3% (95% CI: 45.1 to 51.5%). The anemia prevalence in pregnant women with malaria infection only was 56.0% (95% CI: 50.1 to 61.7%); HIV infection only, 62.5% (95% CI: 25.9 to 89.8%); malaria- HIV coinfection, 60.0 (95% CI: 17.0–92.7%) and without either infection, 44.6% (95% CI: 40.7 to 48.6%). In the fully adjusted models, malaria infection was associated with 27% higher prevalence of anemia (95% CI of prevalence ratio: 1.12 to 1.45; *p* = 0.004), and 3.4 g/L lower hemoglobin concentration (95% CI: - 5.01 to − 1.79; *p* = 0.03) compared to uninfected pregnant women. The prevalence of HIV infection and malaria-HIV coinfection was too low to allow meaningful analysis of their association with anemia or hemoglobin concentration.

**Conclusion:**

Malaria was associated with an increased prevalence of anemia during pregnancy.

## Background

Globally, 38% of pregnant women are anemic, and rates are higher in sub-Saharan Africa at 56%, compared with high income countries at 22% [1]. Anemia in pregnancy is associated with an increased risk of maternal and perinatal mortality and low birth weight [2]. The long-term effects of anemia in pregnancy increase the risk of neurocognitive impairment in the offspring. Two infectious agents, human immunodeficiency virus (HIV) and *Plasmodium falciparum* malaria, are associated with the etiology of anemia in pregnancy in sub-Saharan Africa [3]. In 2018, the prevalence of exposure to malaria infection in pregnancy in sub-Saharan Africa was 29% (equivalence of 11 million pregnancies) [4]. The burden was highest in West and Central African countries. Similarly, the prevalence of HIV infection in pregnant women in sub-Saharan Africa ranges between 11.6 to 22.0% in Southern Africa, 2.2 to 3.9% in Western Africa and Eastern Africa [5]. The prevalence mirrors regional-level HIV prevalence in the general population [6]. The burden of HIV and malaria in this region is one of the leading causes of morbidity and mortality for mothers and their newborns [7–9]. Due to the overlapping geographical distribution of malaria and HIV in sub-Saharan Africa, malaria- HIV coinfection is common and leads to over one million pregnancy complications per year [10, 11]. Such complications include low birth weight, higher rates of neonatal mortality, placental malaria infection, reduced transfer of maternal antibodies and increased risk of mother-child transmission of HIV.

In a recent cross-sectional study aimed at characterizing the prevalence of malaria in people living with HIV, the prevalence of malaria was 7.3% [12]. The prevalence was significantly higher in study participants who did not sleep in insecticide-treated bed nets, participants who were not on co-trimoxazole prophylaxis and those whose CD4 ^+^ T cell count was below 200 cells/μL. The pathological interaction between malaria and HIV in dually infected patients is synergistic and bidirectional [13]. Malaria leads to an increase in HIV viral load and a decline in CD4+ T cell count. Malaria also increases the rate of disease progression from HIV infection to acquired immunodeficiency syndrome. Conversely, HIV contributes to more frequent and more severe malaria infections [14], and an increased risk of congenital infection among pregnant women. People with malaria- HIV coinfection are more likely to harbor parasites at a high density [15, 16]. Immunologically, malaria and HIV both interact with the host’s immune system, leading to complex activation of immune cells and the production of cytokines and antibodies [17]. Therapeutically, HIV impairs the efficacy of antimalarial treatments and may increase adverse events.

Epidemiological studies on the association of malaria- HIV coinfection with anemia in pregnancy are not population-based and suffer from low statistical precision [18, 19]. Therefore, we conducted a large-scale population-based cross-sectional study to explore the association of malaria, HIV and malaria- HIV coinfection, with anemia in pregnancy in sub-Saharan African countries using the most recent Demographic and Health Surveys (DHS) data from 2012 to 2017, and hypothesized that malaria, HIV and malaria- HIV coinfection is associated with anemia in pregnancy in sub-Saharan Africa. A deeper understanding of the epidemiology of the two prevalent and major infections in sub-Saharan Africa and their possible joint effect in contributing to anemia in pregnancy is critical for guiding preventative, control, and treatment strategies to improve fetal, perinatal, and maternal health.

## Methods

### Data sources and participants

Data were from the latest Demographic and Health Surveys from 7 sub-Saharan Africa countries: Burundi, the Democratic Republic of the Congo, Ghana, The Gambia, Mali, Senegal, and Togo (Additional File 1). The data were collected by each country and coordinated by ICF in Rockville, Maryland, USA [20], a global consulting and technology services company that conducts the Demographic and Health Survey funded by the United States Agency for International Development. The survey collected nationally representative health data to monitor and evaluate population health and nutrition programs. The details of sampling methods are discussed elsewhere. (https://dhsprogram.com/What-We-Do/Survey-Types/DHS-Methodology.cfm). In summary, data collection was based on a stratified two-stage probability sampling design, with the samples drawn from the most recent census frame, which is the sampling frame. First, countries were divided into geographic regions. Within these regions, populations were stratified by urban and rural areas of residence. The DHS surveys never define the urban/rural, it always follows the national definition which was defined by the population census. Within these stratified areas, a random selection of enumeration areas (EAs) or primary sampling units (PSUs) were drawn. On average, surveys selected 300–500 EAs with probability proportional to the population [20]. In the second stage of sampling, all households within a PSU were listed from the most recent population census and ~ 30 households per PSU were randomly selected for an interview with the use of equal probability systematic sampling. For each sampled household, women between the ages of 15 and 49 y were sampled. Blood samples were collected from women for laboratory testing of malaria, HIV, and hemoglobin concentration as detailed below.

### Ethical considerations

Each country’s DHS protocols and guidelines, including biomarker collection, were reviewed and approved by their respective Ethical Review Committee and the Institutional Review Board of ICF, USA. Written informed consent was obtained from each participant before the survey. The DHS Program permitted the authors to use the data. The data are entirely anonymous; therefore, the authors did not seek further ethical clearance.

### Assessment of hemoglobin concentration and anemia (outcome)

Blood samples were obtained from a drop of blood taken from a finger prick. A drop of blood from the prick site was drawn into a microcuvette, and hemoglobin analysis was carried out on-site with a battery-operated portable HemoCue analyzer (HemoCue AB, Angelhom, Sweden). Hemoglobin concentrations adjusted for altitude and cigarette smoking status using formulae provided by the US Centers for Disease Control and Prevention [21–23]. Hemoglobin concentration was categorized into anemia levels using World Health Organization standards [24]: severe (hemoglobin concentration < 70 g/L), moderate (hemoglobin concentration is between 70 to 99 g/L), mild (hemoglobin concentration is between 100 g/L and 109 g/L) or any anemia (hemoglobin concentration is < 110 g/L). Respondents received the results of the anemia test immediately, as well as information on how to prevent anemia. Pregnant women with a hemoglobin concentration of < 70 g/L were referred for follow-up care to a local health clinic.

### Exposure and covariates

The primary exposure variables of interest were malaria, HIV and malaria- HIV coinfection. Malaria testing was carried out using SD Pf/Pan Rapid diagnostic tests, Bioline Malaria Ag P.f/Pan (Abbot, Standard Diagnostics, Inc., Gyeonggi-do, and Republic of Korea) which is a qualitative test for the detection of histidine-rich protein II antigen of *Plasmodium falciparum* (Pf), *Plasmodium vivax* (Pv) and other malaria species in human whole blood. HIV testing was carried out using the standard testing algorithm of HIV antibody enzyme-linked immunosorbent assays (ELISAs) [25].: First assay test, Vironostika® HIV Ag/Ab (Biomérieux) ELISA was used. All positives results were subjected to a second ELISA, the Enzygnost® HIV Integral II assay (Siemens). For discordant results, the InnoLia HIV I/II Score (Innogenetics, Ghent, Belgium), was used as a third confirmatory blot assay.

Possible confounders adjusted for were: age, iron supplementation, pregnancy trimesters and intermittent preventative therapy use.

### Statistical analysis

We assumed intra-country and intra-PSU correlation among the responses. Therefore, we fit generalized linear mixed-effects models (GLMM) using the *lme4 package* in R version 3.5.1 [26]. For the binary outcome of anemia (1/0), we used binomial-distributed errors with log link function whereas Gaussian error model and an identity link function was used for average hemoglobin concentration. We employed a log-binomial as opposed to log-odds function to avoid overestimating the prevalence ratio of infection-anemia association due to the high prevalence of anemia in our study population (48%). We accounted for two random effects with PSU nested with country. With the inclusion of country and PSU random effects, we accounted for observable and unobservable factors specific to individuals within a country and PSU such as environmental factors (rainfall, temperature, and altitude) and access to food, which potentially influence hemoglobin concentration [27]. We examined the association of malaria, HIV, and malaria- HIV coinfection, with anemia and hemoglobin concentration in pregnancy using two models. First, in the unadjusted model in which we modeled the association of infection status and anemia status (Hb < 110 g/L). In the second model, a multivariable-adjusted model, we adjusted for the potential confounders listed above. We reported the unadjusted and adjusted prevalence ratios (PR) and 95% confidence intervals for anemia and other covariates included in the model [28]. Also, we reported the estimated means of hemoglobin concentration and the 95% confidence intervals. In both models, we accounted for the random effects of the country and the PSU. We carried out a sensitivity analysis by examining the association of infection status and lower threshold of hemoglobin concentration of < 100 g/L. The analyses were weighted using poststratification fractions defined based on the 2017 population size of the 7 countries. For all the analyses, the significance level was set at α < 0.05. All analyses were performed with R statistical language (R Development Core Team 2017) [29] and map created using ArcGIS version 10.4.1 for desktops (Environmental Systems Research Institute (ESRI), USA.

## Results

### Sample description

**Fig. 1:**
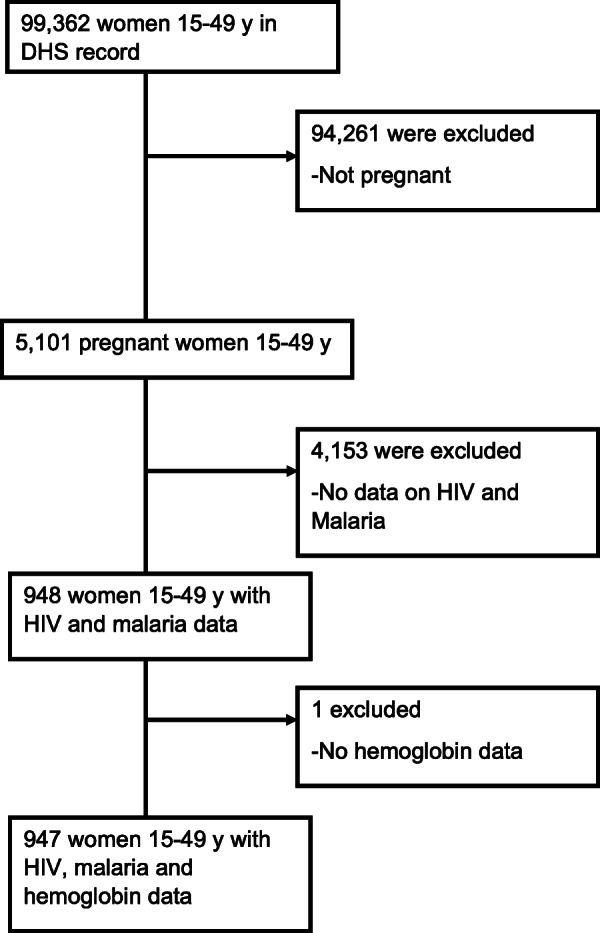
Flow of study participant selection

**Fig. 2:**
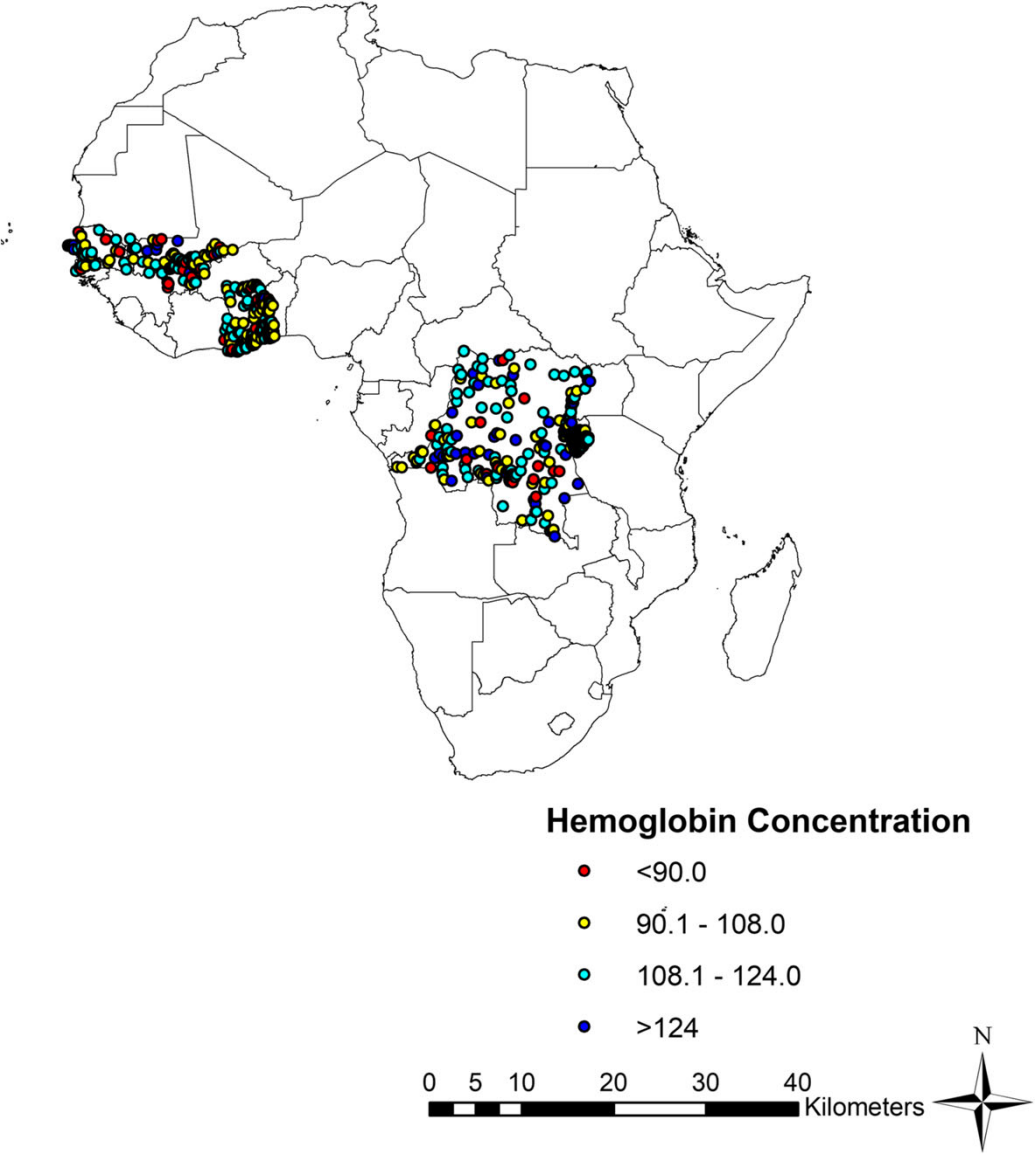
Spatial distribution of hemoglobin concentration for clusters sites (primary sampling units) included in the analysis. Map produced using ArcGIS version 10.4.1 (ESRI, Redlands, CA, United States of America)

**Fig. 3:**
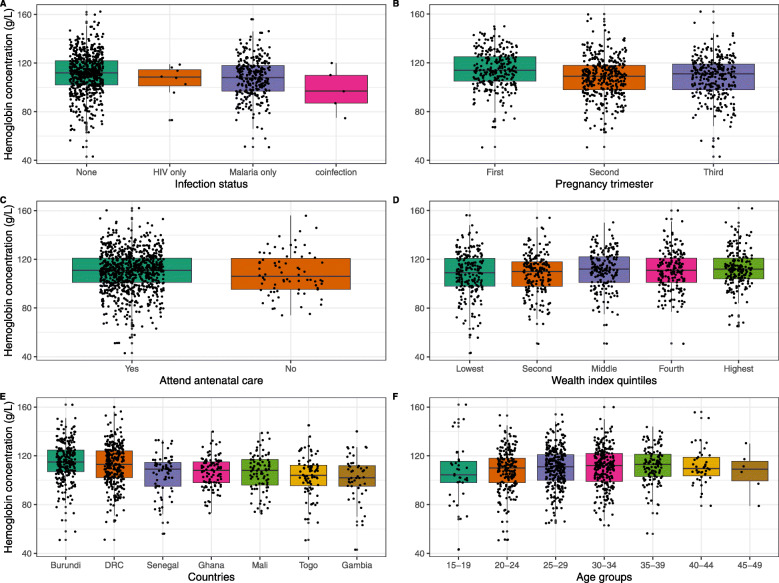
Distribution of hemoglobin concentration. Median, 25th and 75th percentiles of hemoglobin concentrations by infection status (**a**), pregnancy trimester (**b**), antenatal care attendance (**c**), household wealth index quintiles (**d**), countries (**e**), and age group (**f**). Boxplot whiskers form the 1.5x the interquartile range

The flow of study sample selection is shown in Fig. 1**.** Data were collected between November 2012 and December 2017. Detailed in Additional File 1 is a list of the surveys. The sample consisted of 947 pregnant women between the ages of 15–49 years old from 7 different countries (Burundi, the Democratic Republic of the Congo, Gambia, Ghana, Mali, Senegal and Togo). The mean (SD) age of the respondents was 29 (6) years, and half were taking intermittent preventive treatment using sulfadoxine/*pyrimethamine* (Table 1). The proportion of respondents who owned an insecticide treated net was 93% among the overall population. Of all survey respondents, 63% had no formal education, regardless of anemia status, and 67% were from rural residencies. The prevalence of anemia was higher among participants who are adherent to iron supplementation (55% vs. 46%, *p* = 0.01) and in those who were on intermittent preventive treatment with sulfadoxine/*pyrimethamine (*53% vs. 44%, *p* = 0.03). Western African countries had higher prevalence of anemia compared to Central and East African countries. No differences in household wealth quintile status, marital status, breast feeding status and number of births in the previous 5 years. Figure 2 displays the spatial distribution of hemoglobin concentration for clusters sites included in the analysis. In Western Africa, the geographical distribution of low hemoglobin concentration was heterogeneous and was present in large clusters. In Eastern Africa (Burundi), and Central Africa (the Democratic Republic of the Congo), low hemoglobin concentration was homogenously across the region. Figure 3 shows the median (50th percentile), 25th and 75th percentiles of hemoglobin concentration by infection status (A), pregnancy trimester (B), antenatal care attendance (C), household wealth index quintiles (D), countries (E), and age group (F). The overall prevalence of malaria infection only, HIV only and malaria- HIV coinfection was 31% (95% CI: 28.5 to 34.5%, *n* = 293), 1.3% (95% CI: 0.77 to 2.4%, *n* = 13) and 0.52% (95% CI: 0.02 to 1.3%, *n* = 5) respectively. The prevalence of malaria in HIV positive pregnant women was 38% (95% CI: 15 to 68%). Prevalence of any anemia and moderate anemia was higher in pregnant women with malaria and HIV infections than in pregnant women without infections. Mild and severe anemia prevalence was not significantly different across the infection categories (Table 2).

**Table 1:** Background characteristics of the survey participants

Characteristics	All participants (***n*** = 947), No. (%)	Anemia = Yes (***n*** = 458), No. (%)	Anemia = No (***n*** = 489), No. (%)	***p***-value
**Age, mean (SD), y**	28.8 (5.9)	29.0 (6.0)	29.1 (5.8)	0.1202
**Age Categories (y)**				0.6406
15–19	34 (4)	20 (59)	14 (41)	
20–24	204 (22)	105 (51)	99 (49)	
25–29	288 (30)	140 (49)	148 (55)	
30–34	253 (27)	115 (45)	138 (55)	
35–39	120 (4)	53 (44)	67 (56)	
40–44	42 (4)	22 (52)	20 (48)	
45–49	6 (1)	3 (50)	3 (50)	
**Malaria**				**0.0014**
Positive	298 (31)	167 (56)	131 (44)	
Negative	649 (69)	291 (45)	358 (55)	
**HIV infection status**				0.3385
Positive	13 (1)	8 (62)	5 (38)	
Negative	934 (99)	450 (48)	484 (52)	
**Malaria-HIV Category**				**0.001**
Malaria only	293 (31)	164 (56)	129 (44)	
HIV only	8 (0.8)	5 (62.5)	3 (37.5)	
Malaria-HIV coinfection	5 (0.5)	3 (60)	2 (40)	
No infection	641 (68)	286 (45)	355 (55)	
**Hemoglobin mean (SD), g/L,**	11.0 (1.7)	9.7 (1.2)	12.2 (1.0)	**< 0.0001**
**Insecticide Treated Nets**				0.8073
Yes	503 (93)	249 (49.5)	254 (50.5)	
No	40 (7)	19 (47.5)	21 (52.5)	
**Intermittent Preventive Treatment**				**0.003**
Yes	453 (48)	242 (53)	211 (47)	
No	487 (52)	213 (44)	274 (56)	
**Education level**				0.55
No formal schooling	348 (37)	175 (50)	173 (50)	
Primary	322 (34)	159 (49)	163 (51)	
Secondary	261 (28)	117 (45)	144 (55)	
More than secondary	16 (2)	7 (44)	9 (56)	
**Household wealth quintile**				0.43
Lowest	210 (22)	110 (52)	100 (48)	
Second	180 (19)	90 (50)	90 (50)	
Middle	189 (20)	86 (45.5)	103 (54.5)	
Fourth	183 (19)	91 (50)	92 (50)	
Highest	185 (20)	81 (44)	104 (56)	
**Place of residence**				0.35
Urban	316 (33)	146 (46)	170 (54)	
Rural	631 (67)	312 (49)	319 (51)	
**Iron Supplementation**				**0.01**
Less than 90 days	714 (75)	329 (46)	385 (54)	
Equal to or greater than 90 days	233 (25)	129 (55)	104 (45)	
**Marital Status**				0.13
Not married	9 (1)	2 (22)	7 (78)	
Married	913 (96)	447 (49)	466 (51)	
Divorced	25 (3)	9 (36)	16 (64)	
**Births in the last 5 years**				0.88
2 or less	913 (96)	442 (48)	471 (52)	
More than 2	34 (4)	16 (47)	18 (53)	
**Currently Breastfeeding status**				0.96
Yes	105 (11)	51 (49)	54 (51)	
No	842 (89)	407 (48)	435 (52)	
**Country**				**0.0007**
Burundi	246 (26)	103 (42)	143 (58)	
The Democratic republic of the Congo	302 (32)	127 (42)	175 (58)	
Ghana	107 (11)	58 (54)	49 (46)	
The Gambia	49 (5)	31 (63)	18 (37)	
Mali	88 (9)	50 (57)	38 (43)	
Senegal	71 (8)	38 (54)	33 (46)	
Togo	84 (9)	51 (61)	33 (39)

**Table 2:** Estimated percentages and 95% confidence interval of distribution of outcome variables across exposure categories

Anemia severity group % (95% CI)	Total n = 947	No infection ***n*** = 641	Malaria only n = 293	HIV only ***n*** = 8	Malaria- HIV coinfection, n = 5
Any Anemia	48 (46–52)	45 (41–49)	56 (50–62)	63 (26–90)	60 (17–93)^a^
Mild	23 (20–25)	21.8 (19–25)	25 (20–30)	38 (10–74)	0 (0–54)
Moderate	24 (21–27)	20 (17–23)	29 (24–35)	25 (5–65)	60 (18–92)^a^
Severe	2 (1–3)	2.0 (0.8–2.9)	2 (1–4)	0.0 (0–0.4)	0.0 (0–54)

*HIV* Human Immunodeficiency Virus

^a^ Proportions in the 4 groups were significantly different from each other, *P* < 0.05 (Fisher Exact probability test)

### Association between the infection status and anemia in pregnancy

Table 3 shows the unadjusted and adjusted PRs of the association between infection categories and anemia in pregnant women. In model adjusted only for country and PSU random effects (unadjusted model), malaria, HIV and malaria- HIV coinfection were associated with 26, 42 and 36% higher prevalence of anemia compared to uninfected pregnant women. In model adjusted for a full set of covariates (adjusted model) and country and PSU random effects, malaria was associated with 27% higher prevalence of anemia, HIV with 41% higher prevalence of anemia, and malaria- HIV coinfection was associated with 22% higher prevalence of anemia than pregnant women without either infection. In the fully adjusted model, anemia prevalence was higher in the second and third trimesters than in the reference category of pregnant women in the first trimester. Malaria and HIV infections explained 6% of the variance in anemia and hemoglobin concentration.

**Table 3:** Unadjusted and multivariable-adjusted prevalence ratio (95% confidence interval) for the association of infection status with anemia (Hb < 110 g/L) in pregnant women, 15–49 y old^a^

	Unadjusted PR (95% CI)	***p***-value	Adjusted PR (95% CI)	***p***-value
**Infection status**				
Malaria only	1.26 (1.12–1.45)	0.0006	1.27 (1.12–1.45)	0.0004
HIV only	1.42 (0.80–2.41)	0.20	1.41 (0.83–2.41)	0.21
Malaria- HIV coinfection	1.36 (0.66–2.74)	0.40	1.22 (0.61–2.46)	0.58
No infection	Reference		Reference	

^a^Associations were estimated with the use of generalized linear mixed-effects models (binomial-distributed errors with log link function). All values are PRs (95% CIs)

In Table 4, we report estimated means results of hemoglobin concentration (g/L) as a continuous dependent variable. Compared to the participants without any infections, malaria infection was associated with a 3.4 g/L lower hemoglobin (95% CI: - 5.01 to − 1.79; *p* = 0.03, Fig. 3a). Results for HIV and malaria- HIV coinfection were nonsignificant (*p* = 0.84, and *p* = 0.35 respectively). Compared to the first trimester (*n* = 277), the mean hemoglobin concentration was lower in participants in the second (*n* = 369) and third trimester (*n* = 301) (Fig. 3**b**). In a sensitivity analysis examining the association of infection status and lower threshold of hemoglobin concentration of < 100 g/L, the results are consistent with the primary model and are reported in Additional File 2**.**

**Table 4:** Associations between the infection status and anemia in pregnant women, 15–49 y old with the hemoglobin concentration (in g/L) as continuous dependent variables^a^

Infection status	Adjusted estimated hemoglobin means (95% CI)	estimates (SE)	***p***-value
Malaria only	106 (100.1 to 113)	−3.4 (1.6)	0.03
HIV only	109 (95.4 to 122)	−1.2 (6.1)	0.84
Coinfection	102 (83.7 to 120)	−8.1 (8.7)	0.35
No infection	110 (103.6. to 116)	**Reference**	

^a^Associations were estimated with the use of generalized linear mixed-effects models (Gaussian error model and an identity link function). 95% of SEs were clustered by country and primary sampling units. Adjusted for age, trimesters and iron supplementation

## Discussion

We showed that malaria infection had a significant association with anemia and lower hemoglobin concentration in pregnancy. The finding was robust to adjustment for various confounding factors. The absence of any association for HIV and malaria-HIV coinfection is possibly due to low statistical precision. The quantitatively small variation of 6% in hemoglobin concentrations and anemia explained by malaria and HIV infections shown in our analysis suggests that in sub-Saharan Africa, anemia in pregnancy is a result of a complex interplay of other factors in addition to malaria and HIV. Such factors include; nutritional (such as iron and folate deficiency) and genetics (such as inherited hemoglobin disorders) and inflammation [4, 30–32].

The high prevalence of anemia in pregnant women was consistent with a previously reported prevalence of 56% in studies of the same region [1]. The association of anemia in pregnant women with either HIV or malaria separately or both has been reported in smaller studies (and reviews) in Africa [33, 34]. The lack of a significant association with HIV and malaria-HIV coinfection should be interpreted with caution. Due to lower numbers in these groups, the standard errors were large providing a wide confidence interval. However, the malaria-HIV coinfection group had the lowest point estimates of hemoglobin concentration compared to either malaria or HIV infection alone, suggesting a possible joint effect between malaria and HIV (Fig. 3 A). The prevalence of malaria in HIV infected pregnant women was 7 percentage points higher than in the pregnant women without HIV infection. This is agreement with a prospective study in South Africa, in which HIV-infected adults were at increased risk of severe malaria [35].

The mechanisms by which malaria and HIV lead to anemia in pregnancy are complex. Malaria infection during pregnancy causes acute anemia mainly through direct destruction of erythrocytes, sequestration of infected and uninfected erythrocytes by the spleen, and decreased erythropoiesis. Furthermore, the placental sequestration of malaria-infected erythrocytes is typical in pregnant women [36], and occurs in the intervillous space. Placental sequestration is aided by placental adhesion molecules particularly chondroitin sulfate A [37] which interacts with the *Plasmodium falciparum* erythrocytes membrane protein-1,VAR2CSA [38]. Hepcidin, the master regulator of bioavailable iron and a key mediator of anemia, is elevated in placental-malaria [39, 40]. In addition to increasing the susceptibility of pregnant women to anemia, hepcidin is causally associated with anemia or lower hemoglobin concentration in the offspring [40], suggestion long-term effects of malaria in pregnancy in infants. The resultant mechanical vascular blockage by placental sequestration impairs placental blood flow to the fetus, consequently increasing the risk of intrauterine growth restriction and fetal demise [38]. On the other hand, HIV in pregnancy leads to chronic anemia, which develops as a result of HIV-induced inflammation, reduced food intake, and impaired iron absorption caused by antiretroviral therapy [41].

Preventative strategies for anemia in pregnancy due to malaria and HIV are well documented. The current guidelines for the management of HIV in pregnant women include the use of trimethoprim-sulfamethoxazole (co-trimoxazole) in addition to antiretroviral therapy, which both lower odds of coinfection [42]. In a prospective study in Uganda, a combination of co-trimoxazole, antiretroviral therapy, and insecticide-treated bednets substantially reduced the frequency of malaria in adults with HIV [43]. Malaria control in pregnant women can be achieved chiefly by vector control using insecticide treated bednets, residual spraying and use of intermittent preventive treatment in pregnancy with sulfadoxine/*pyrimethamine*. Unexpectedly however, in our study, intermittent preventive treatment with sulfadoxine/*pyrimethamine* was associated with deleterious effects on maternal hemoglobin. Such a finding could be explained by increasing parasite resistance in various parts of sub-Saharan Africa resulting to the effect of IPTp transitioning from net benefit to neutral or net harm [44]. There is however a dilemma of using intermittent preventive treatment in pregnant women who are on antiretroviral therapy and co-trimoxazole prophylaxis for opportunistic infections due to the risk of sulfonamide-induced adverse drug reactions. Therefore, intermittent preventive treatment is contraindicated in this group (see an excellent review by Tebit Kwenti) [13].

Although we observed an association between malaria infection and anemia in pregnancy, the variance in hemoglobin concentration and anemia explained by this association was small (6%), suggesting that other factors other than malaria are attributable to the high prevalence of anemia in pregnant women. In sub-Saharan Africa, low hemoglobin concentration is also caused by nutritional deficiencies of iron [45], vitamin B12 and folate [46]; genetic traits such as sickle cell trait [32]; parasitic infections such as hookworm infection [47], and schistosomiasis [48] and should be explored in pregnant women presenting with anemia. Intervention studies demonstrate the positive effect of iron supplementation on hemoglobin concentrations in those with anemia, both in pregnant [49] and non-pregnant women [50, 51]. However, in our analysis, adherence to iron supplementation (defined as using iron supplementation for ≥90 d during pregnancy) was low at 25%, consistent with our recent findings [51], and was not significantly associated with protection against anemia in pregnancy.

Factors contributing to the mean hemoglobin differences between the Western region and the rest of sub-Saharan Africa have been explored. Geospatial analysis of hemoglobin concentration in sub-Saharan Africa concluded that known environmental drivers of anemia-causing parasitic infections and nutritional iron deficiency, such as distance to a perennial water body, land surface temperature and normalized difference vegetation index show stronger effects for Western Africa than Eastern Africa [27]. In addition, inherited hemoglobin disorders particularly in Western Africa, could, in part, account for the difference anemia variation between Western and Central and East Africa [32].

Our study had some limitations. First, the cross-sectional nature of the study could not allow us to infer causality. Anemia is a chronic multifactorial syndrome and women are often anemic before becoming pregnant. Therefore, the temporal association between malaria during pregnancy and anemia occurrence could not be established in our study. Second, a onetime assessment of malaria and HIV in this cross-sectional study design could have led to an underestimation of the infection rate since infection testing was not repeated. Third, pregnancy is self-reported hence early pregnancy is likely to be under reported. The pregnancy reporting behavior from the DHS data shows that only 75% of women with a 10-week pregnancy report that they are pregnant. Fourth, variable selection for the infection-anemia models was mainly driven by information availability from the survey datasets. Consequently, other possible confounding variables such as gestational age, nutritional status (pre-pregnancy BMI, gestational weight gain) and helminths that were not adjusted in our analysis could have biased the observed association. Nevertheless, considering we adjusted for major possible confounders known to be associated with malaria and HIV and anemia, our study provides stable estimates of anemia in pregnancy in several countries in sub-Saharan Africa using a nationally representative sample. Such factors include pregnancy trimester, iron supplementation, and intermittent malaria preventive treatment use. Lastly, our study had low number of coinfected pregnant women resulting in excessively wide confidence intervals. The low prevalence of 1.3% in the study population is explained by the low prevalence of HIV in Western, Central and Eastern Africa, which represents our study sample. It is likely such a selection bias could have influenced the estimates towards the null. Consequently, our findings may not generalize to entire sub-Saharan Africa.

## Conclusion

Malaria was associated with an increased prevalence of anemia during pregnancy. The prevalence of HIV infection and malaria-HIV coinfection was too low to allow meaningful analysis of their association with anemia or hemoglobin concentration.

## Supplementary information

**Additional File 1.** Country- specific sample size and population. Sample size summary for the analyzed DHS data indicating the country, year of survey, number of pregnant women, region and 2017 population size.

**Additional File 2.** Sensitivity analysis. Association of infection status with anemia (Hb < 100 g/L) in pregnant women, 15–49 y old.

## Data Availability

The data sets were accessed after obtaining permission from Measure DHS. Data sets under the project Malnutrition in Africa. The analyzed dataset is available from: https://dhsprogram.com/data/available-datasets.cfm. Data is available for download from the website after acquiring permission from Measure DHS. We confirm that we did not have special access privileges to this data.

## Abbreviations

HIV: Human immunodeficiency virus
DHS: Demographic and health survey
USAID: United States Agency for International Development
PR: Prevalence Ratio
Hb: Hemoglobin
