# Supplementary material for: Associations of malaria, HIV, and coinfection, with anemia in pregnancy in sub-Saharan Africa: a population-based cross-sectional study
Source: BMC Pregnancy Childbirth. 2020 Jun 29;20:379. doi: 10.1186/s12884-020-03064-x (PMC7324981; doi:10.1186/s12884-020-03064-x)
Supplement: Supplementary file 1 — Additional File 1. Country- specific sample size and population. Sample size summary for the analyzed DHS data indicating the country, year of survey, number of pregnant women, region and 2017 population size. [file 12884_2020_3064_MOESM1_ESM.docx]

| **Country** | **Year** | **No. of pregnant women** | **Region** | **Population**  **(thousands)** |
| --- | --- | --- | --- | --- |
| Burundi | Oct 2016- Feb 2017 | 246 | East Africa | 10, 864 |
| The Democratic Republic of the Congo, | Aug 2013- Feb 2014 | 302 | Central Africa | 81, 340 |
| The Gambia | Feb -April 2013 | 49 | West Africa | 2,101 |
| Ghana | Sep-Dec 2014 | 107 | West Africa | 28, 834 |
| Mali | Nov 2012- Jan 2013 | 88 | West Africa | 18, 542 |
| Senegal | Apr-Dec 2017 | 71 | West Africa | 15, 851 |
| Togo | Nov 2013-April 2014 | 84 | West Africa | 7, 798 |

**Country- specific sample size and population.** Description of data: Sample size summary for the analyzed DHS data indicating the country, year of survey, number of pregnant women, region and 2017 population size.
