# Supplementary material for: Associations of malaria, HIV, and coinfection, with anemia in pregnancy in sub-Saharan Africa: a population-based cross-sectional study
Source: BMC Pregnancy Childbirth. 2020 Jun 29;20:379. doi: 10.1186/s12884-020-03064-x (PMC7324981; doi:10.1186/s12884-020-03064-x)
Supplement: Supplementary file 2 — Additional File 2. Sensitivity analysis. Association of infection status with anemia (Hb < 100 g/L) in pregnant women, 15–49 y old. [file 12884_2020_3064_MOESM2_ESM.docx]

**Sensitivity analysis:** Association of infection status with anemia (Hb< 100 g/L) in pregnant women, 15-49 y old.

|  | **Unadjusted PR (95% CI)** | **p values** | **Adjusted PR (95% CI)** | **p values** |
| --- | --- | --- | --- | --- |
| **Hemoglobin <100 g/L** |  |  |  |  |
| Malaria only | 1.37 (1.04-1.79) | 0.02 | 1.33 (1.02-1.80) | 0.004 |
| HIV only | 1.17 (0.19- 3.66) | 0.82 | 1.21 (0.21-3.72) | 0.78 |
| MHC | 2.81 (0.69-7.41) | 0.08 | 2.41 (0.60-6.40) | 0.13 |
| No infection | Reference |  | Reference |  |
